# Pre-hospital triage of suspected acute stroke patients in a mobile stroke unit in the rural Alberta

**DOI:** 10.1038/s41598-021-84441-0

**Published:** 2021-03-02

**Authors:** Mahesh P. Kate, Thomas Jeerakathil, Brian H. Buck, Khurshid Khan, Ali Zohair Nomani, Asif Butt, Sibi Thirunavukkarasu, Tomasz Nowacki, Hayrapet Kalashyan, Mar Irida Lloret-Villas, Atlantic D’Souza, Sachin Mishra, Jennifer McCombe, Kenneth Butcher, Glen Jickling, Maher Saqqur, Ashfaq Shuaib

**Affiliations:** 1 Clinical Neurosciences, Edmonton Zone, Alberta Health Services, Edmonton, Canada; 2 Division of Neurology, Department of Medicine, University of Alberta, Edmonton, Canada; 3 Clinical Neurosciences, Central Zone, Alberta Health Services, Red Deer, Canada; 4 Department of Clinical Neurosciences, Prince of Wales Clinical School, Randwick, Australia

**Keywords:** Cerebrovascular disorders, Stroke

## Abstract

Mobile Stroke Unit (MSU) expedites the delivery of intravenous thrombolysis in acute stroke patients. We further evaluated the functional outcome of patients shipped to a tertiary care centre or repatriated to local hospitals after triage by MSU in acute stroke syndrome in rural northern Alberta. Consecutive patients with suspected acute stroke syndrome were included. On the basis of neurology consultation and, Computed Tomography findings, patients, who were thrombolysed or needed advanced care were transported to the Comprehensive stroke center (CSC) (Triage to CSC group). Other patients were repatriated to local hospital care (Triage to LHC group). A total of 156 patients were assessed in MSU, 73 (46.8%) were female and the mean age was 66.6 ± 15 years. One hundred and eight (69.2%) patients, including 41 (26.3%) treated with thrombolysis were transported to the CSC (Triage to CSC group) and 48 (30.8%) were repatriated to local hospital care. The diagnosis made in MSU and final diagnosis were matching in 88% (95) and 91.7% (44, p = 0.39) in Triage to CSC and Triage to LHC groups respectively. Prehospital triage by MSU of acute stroke syndrome can reliably repatriate patients to the home hospital. The proposed model has the potential to triage patients according to their medical needs by enabling treatment in home hospitals whenever reasonable.

## Introduction

The benefits of reperfusion therapies in acute ischemic stroke decrease over time with the best outcomes seen with early initiation of treatment^1,2^. To expedite the evaluation of acute suspected stroke patients, several health regions including one in Canada (Edmonton, Alberta) have added a mobile stroke unit (MSU) to their acute stroke program. MSUs allow for the prehospital evaluation and ultra-early fibrinolytic treatment of ischemic stroke patients. Studies have shown that MSUs decrease stroke onset to treatment times with IV tPA, and increase the percentage of patients receive reperfusion treatment^1,3–5^ and the number of patients being discharged home^6,7^.

While the initial focus of MSUs has been on the earlier delivery of IV tPA, it is increasingly being recognized that MSUs can play a central role in triaging suspected stroke patients for EVT. Many suspected strokes patient will need to be screened to identify the small group who are likely to benefit from EVT (more than 7798 acute stroke syndrome patients screened 13% receive IV Thrombolysis and < 1% undergo mechanical thrombectomy in EXTEND IA study)^8,9^. Screening for EVT in many Canadian regions involves prehospital diversion to a tertiary hospital. As a result, many tertiary centres have experienced exponential increases in stroke volumes over the past few years.

The main aim of this study is to determine the outcome of patients shipped to a comprehensive stroke centre or repatriated to local hospitals after triage by MSU in acute stroke syndrome in rural northern Alberta.

## Results

### Acute reperfusion therapy

During the study period (Feb 2017–Nov 2019), the MSU was dispatched 156 times within the catchment area after consultation with emergency department physicians in rural hospitals. The mean age was 66.6 ± 15 years and 46.8% (73) were females. There were 77 (49.4%) patients with acute ischemic stroke, 7 (4.5%) with ICH, 19 (12.2%) patients with transient ischemic attacks and 53 (34%) patients with stroke mimics. From these 156 patients, 41 (26.3%) patients received thrombolysis with alteplase in the MSU within the time window, including 37 (90.2%) with acute ischemic stroke and 4 (9.8%) stroke mimics. Patients with an MSU diagnosis of acute ischemic stroke that were not thrombolysed included 39 (n = 109, 35.8%) patients with minor stroke or TIA and 29 (26.6%) patients that were outside thrombolysis window. In patients who received intravenous thrombolysis the median last seen well to patient-contact time was 154 (110–209) min, median patient-contact to thrombolysis time was 21 (19–27) min and median CT to thrombolysis time was 11.5 (9–15) min. Fourteen (n = 156, 9%) patients received mechanical thrombectomy, 6 (n = 14, 42.8%) patients received direct mechanical thrombectomy without IV thrombolysis. None of the patients who received thrombolysis developed secondary hemorrhage.

### Triage to LHC group

Forty-eight (n = 156, 30.8%) patients were repatriated to home hospital, most of them were either stroke mimic (n = 21, 43.7%) or TIA (n = 14, 29.2%) (Table 1). The patients in the Triage to LHC group” were likely to have lower NIHSS, higher dispatch to patient time and more likely to be TIA. With low numbers there were no statistical difference in the baseline clinical, imaging work flow times, etiological investigation and follow-up characteristics between the Triage to CSC and Triage to LHC groups (Table 1). During follow-up evaluation, the final diagnosis was similar in the Triage to CSC and Triage to LHC groups. A total of 5 (n = 103, 4.9%) patients developed recurrent stroke during follow-up. These included 3 (2.8%) in the Triage to CSC group and 2 (4.2%) in the Triage to LHC group. One patient repatriated to home hospital developed early neurological deterioration had to be transported to the tertiary care center.

**Table 1:** Characteristics of acute stroke syndrome patients evaluated in the mobile stroke unit in triage to local hospital care and triage to comprehensive stroke centre groups.

	Triage to LHC (n = 48)	Triage to CSC (n = 108)	p value
Mean ± SD age, years	70 ± 12.7	66.4 ± 15.7	0.16
Sex, female:male	28:20	45:63	0.058
Median dispatch to patient-contact, min	59 (34–81)	42 (26–62)	0.016
Median symptom onset to patient-contact, min	184 (134–230.5)	153(84–210)	0.11
Median NIHSS in ambulance	1 (0–2.5)	5(2–11)	< 0.0001
Median symptom onset to CT, min	193 (149–283)	165 (104–217)	0.19
Median patient-contact to CT, min	11 (10–18)	12 (9.5–15)	0.65
Mean systolic BP, mmHg	146.6 ± 27.4	149.3 ± 23.1	0.6
Mean diatsolic BP, mmHg	78.7 ± 15.3	84.7 ± 15.1	0.08
Hypertension, n (%)	24 (50)	65 (60.2)	0.29
Diabetes, n (%)	10 (20.8)	29 (26.8)	0.54
Dyslipidemia, n (%)	19 (39.6)	39 (36.1)	0.72
Atrial fibrillation, n (%)	10 (20.8)	17 (15.7)	0.49
Coronary artery disease, n (%)	7 (14.6)	16 (14.8)	1
Smoking, n (%)	5 (10.4)	26 (24.1)	0.053
Past history of TIA or stroke, n (%)	11 (22.9)	25(23.1)	0.9
Diagnosis: stroke, n (%)	13 (27.1)	71 (65.7)	< 0.0001
TIA, n (%)	14 (29.2)	5 (4.6)	
Stroke mimic, n (%)	21 (43.7)	32 (29.6)	
Matching diagnosis (ambulance and final diagnosis), n (%)	44 (91.7)	95 (88)	0.39
Vascular imaging performed, n (%)	29 (60.4)	83 (76.8)	0.053
Holter monitoring performed or atrial fibrillation known, n (%)	16 (33.3)	52 (48.1)	0.11
Recurrent stroke, n (%)	2 (4.2)	3 (2.8)	0.64
Median modified Rankin scale	0 (0–3)	2(0–4)	0.53
Mortality, n (%)	8 (16.7)	14 (13)	0.9

### Stroke mimics

**Figure 1:**
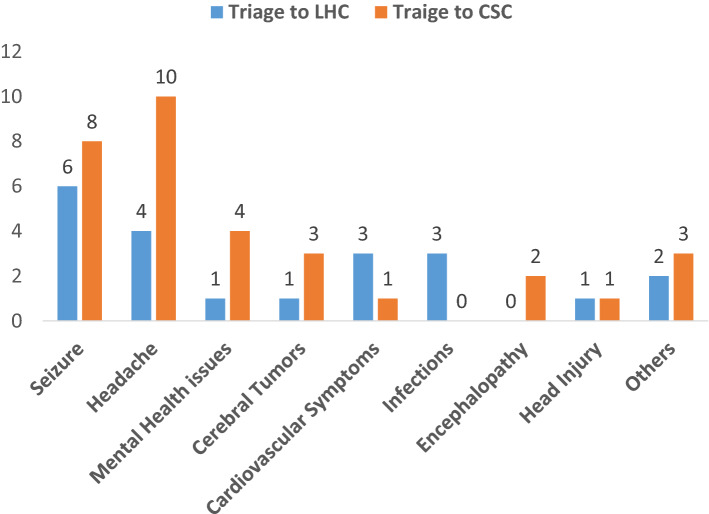
Distribution of Patients with a Stroke mimic. Distribution of patients with a Stroke mimic in the repatriated and shipped groups. Seizure and headache were the most common mimic presentations.

Fifty-three (34%) patients received a final diagnosis of a stroke mimic. Thirty-two (60.4%) patients with a diagnosis of stroke mimic were triaged to CSC and the remainder 21 (39.6%) patients were triaged to LHC. Among the patients with stroke mimics, 14 (26.4%) had seizures, 14 (26.4%) had acute headaches, five (9.4%) patients had mental health issues including conversion disorder, four (7.5%) patients had cerebral tumors, four (7.5%) patients had cardiovascular symptoms including syncope or malignant hypertension, three (5.7%) patients had systemic infections, two (3.8%) patients had encephalopathy and head-injury each (Fig. 1).

## Discussion

The MSU is now an established means to assess patients with acute ischemic stroke syndrome and deliver in field intravenous thrombolysis when appropriate^1,3–5^. Imaging excludes ICH and where CT angiography is available, allows for the detection of proximal intracranial vascular occlusion. There is considerable evidence from Europe, North America and Australia that treatment of acute stroke in an MSU can increase the number of patients treated within the “golden hour” from onset^10–15^. In addition to possible better outcome, there is also no increased risk of hemorrhagic transformation or other complications. The experience to date has mostly been in urban settings^11,15^.

We describe our results with MSU in a program based in a rural setting in Alberta, Canada. Similar to previous reports, we show that there is a rapid “door to treatment time” in patients in whom thrombolysis is offered in the ambulance (median 21 min). In our study the ambulance is being dispatched upto a radius of 250 km, due to small current numbers the validity of such long distance dispatch needs further confirmation. Our thrombolysis rates are comparable to reports in the literature. Although complications are reported in the transfer of patients treated with rt-PA particularly with basilar thrombosis^16^, we did not experience any complications during transfer in patients in whom Alteplase was transfused.

During the three years, we were consulted on 156 patients in the MSU in rural settings. Following neurological consultation and imaging, 34% of the patients were repatriated back to the referring hospital. Most of these patients were stroke mimics or TIA/minor stroke. A proportion of similar patients were shipped to the tertiary care center. During follow-up, complications including new ischemic events were similar between patients repatriated and those transferred to the CSC vis MSU. Arguably, two-third of patients examined and imaged in the MSU, mostly TIA and minor stroke and stroke mimics can be repatriated to the rural referral hospital following the ambulance consultation. This repatriation is particularly important in Canada as it allows for a reduction of unnecessary referrals to overcrowded emergency departments across Canada. The MSU thus, in addition to allowing for early thrombolysis, also offers an important venue for urgent neurological consultation in the field.

Unlike reports within urban settings where the MSUs are activated by the EMS dispatcher with frequent ‘stood-downs’ for non-stroke related events, the majority of activations in the rural settings are from family doctors or other emergency department consultants once the patients had been assessed in the local hospital^10,17^. The number of false activations were therefore very low in our study. Most patients had mild symptoms secondary to an ischemic stroke or were stroke mimics. There is recent evidence on the reliability of the diagnosis of TIAs in the prehospital settings^18^. In this study 1081 patients were assessed in 8 emergency departments in Sweden, 680 were diagnosed as stroke or TIA, the likelihood for diagnosis of stroke or TIA increased with increasing age, previous history of myocardial infarction or stroke, motor weakness or speech disturbances and high systolic blood pressure^18^. In a recent report from Berlin, Germany a telestroke mimic (TM) score was used to differentiate patients with ischemic stroke (423 patients) from mimics (124 patients). A six-item score correctly identified mimics in 74% of times in an urban setting (TM score < 16). Identification was helpful with appropriate hospital destination thus avoiding unnecessary traffic to stroke centers in the city^19^. The percentage of suspected acute stroke patients with a final diagnosis of ‘stroke mimics’ was 34% in our study and is within the range of between 30 and 50% reported in the literature^20^. Three-fourths of patients with suspected diagnosis of ‘stroke mimics’ were correctly identified in the MSU following assessment and imaging.

There is a limited non-stroke patient experience in the MSU. A single additional report discussed the successful use of MSU to assess a patient with suspected brain trauma^21^. Our study suggests that the mobile CT unit can potentially have expanded use, especially in the rural setting where smaller pockets of populations with limited access to diagnostic imaging.

A small sample size is the primary limitation of our study. Nevertheless, our study has several strengths. The MSU at UAH caters rural catchment area. Our MSU had a stroke fellow on board which increased the likelihood of complete neurological examination. Recent studies have shown that the presence of good tele-neurology system may help mitigate the need for a neurologist onboard. Over 3 years we triaged 156 patients with MSU, with average activation of 52 per year. This is far less than the actual potential of the MSU in Alberta. The reason for less activation could be lack of awareness in the rural physicians about the criteria for activation the MSU program.

MSU is a reliable mode of delivering field thrombolysis in an expedited manner. However, it can also be utilized as field-triage for other acute neurological presentations including seizure, headache and head-injury. This approach may make the MSU a sustainable cost-effective strategy for screening patients and appropriate local treatment in addition to thrombolysis. This may help reduce the burden of emergency departments and stroke units.

## Methods

### Design

The ACHIEVE (AmbulanCe Housed Ischemic Stroke trEatment with intraVEnous Thrombolysis) is a prospective study where the MSU was deployed to meet up with inbound ambulances that transported suspected acute stroke patients from hospitals surrounding the city of Edmonton, Alberta within a radius of 250 km^22^. The project became operational in February of 2017. The MSU was available between 8 am and 4 pm for 5 days a week (Monday–Friday). The University of Alberta Ethics Review Committee approved the project. All study procedures were in accordance to the provincial regulations and ethical guidelines. An informed consent was obtained from all the patients or the surrogate decision makers. The University of Alberta Hospital (UAH) is the only thrombectomy-capable hospital and one of two hospitals that offer thrombolysis to eligible acute stroke patients in Edmonton zone. It serves the population of Northern Alberta and is linked to 14 CT-equipped emergency departments across a large catchment area via telemedicine. Despite the telestroke link, a large geographic region still has very patchy acute stroke coverage. Patients in rural emergency departments where CT scans are not available have to be transferred to the UAH for diagnosis and reperfusion therapies. The “drip-and-ship” method transfers patients to the UAH if they are potential candidates for clot retrieval. With the introduction of the MSU, the acute stroke coverage was re-organized so that patients from hospitals within a 250 km radius were now candidates for treatment in the field with methods described above^22,23^.

### Mobile stroke unit

The MSU is a custom-built ambulance (Demers, Beloeuil, Quebec, CA) with a portable CT scanner (CereTom, Samsung, Boston, MA, USA), telestroke equipment (Lifebot, Phoenix, Arizona, US) and ‘point-of care’ laboratory to measure blood count and INR (POCHi, Sysmex Canada). The MSU is based at the UAH emergency area. The specialized MSU team consists of a stroke fellow, CT technologist, registered nurse, primary care paramedic, and advanced care paramedic. The CT images are transferred to the vascular neurologist at the UAH via a wireless network in a Picture Archiving and Communication System (PACS). The point-of-care laboratory is able to measure the blood glucose, hemoglobin levels, platelet and leukocyte count and the international normalized ratio (INR). In addition, the MSU carries Alteplase, anti-hypertensive medications and other medications that may be required for emergency care or intubation in the field^22^.

### Study procedures

**Figure 2:**
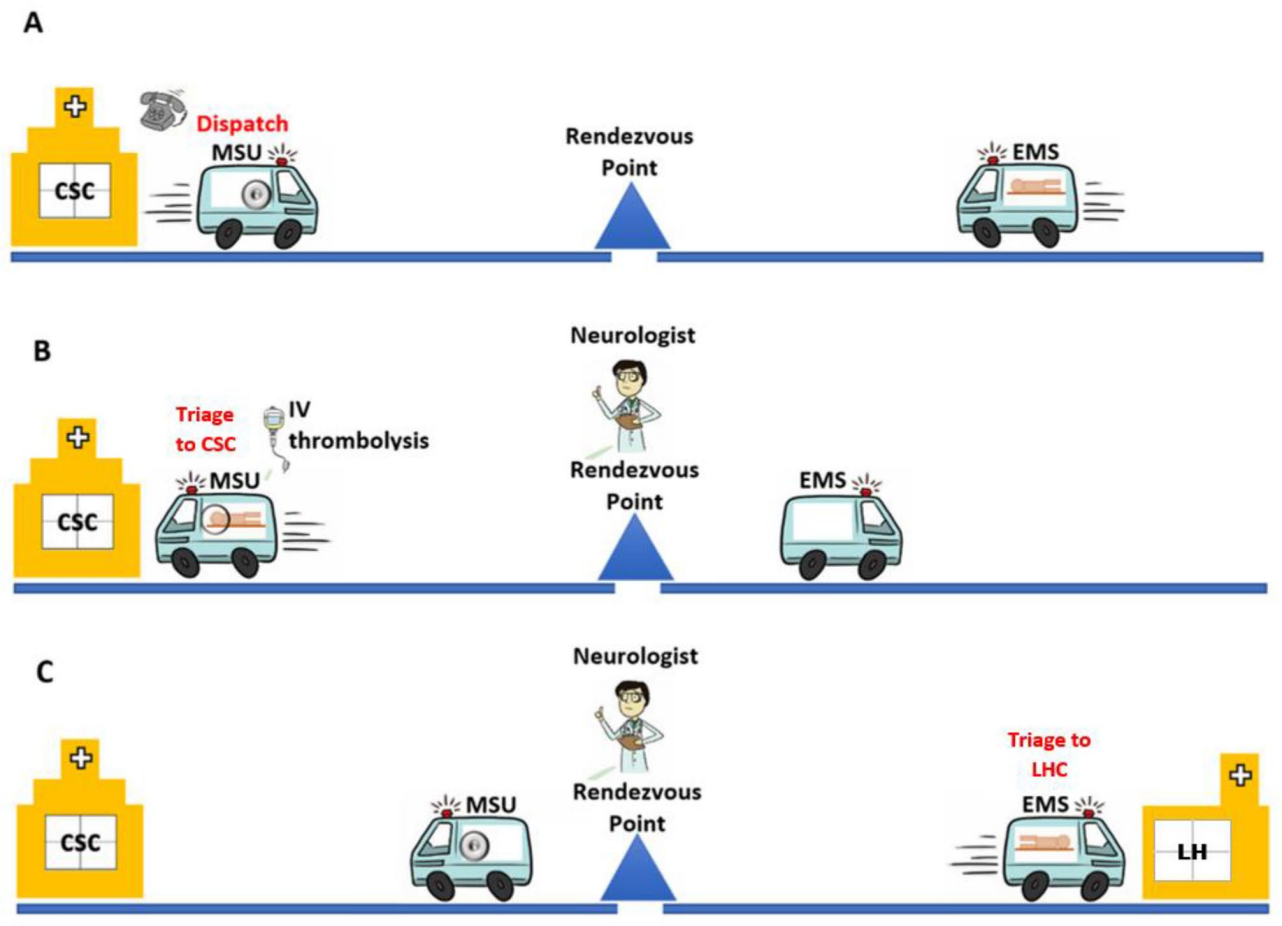
Dispatch of Mobile Stroke Unit and Transportation of Patients. **(A)** Mobile Stroke Unit (MSU) was dispatched from comprehensive stroke center (CSC) to meet Emergency Medical services (EMS) ambulance at a Rendezvous point in the field usually midway between travel. **(B)** If the clinical assessment by Stroke Fellow and imaging suggests acute stroke requiring thrombolysis or need of advanced care, the patient was transported to CSC with possible en-route IV thrombolysis. **(C)** If the clinical assessment by Fellow and imaging was suggestive of minor stroke or a stroke mimic not requiring advanced care, the patient was transported to the nearest home hospital (HH) or local hospital care.

The operational design for deployment of the MSU for rural evaluations is shown in Fig. 2. In brief, the UAH telestroke neurologist dispatches the MSU after a telephone consultation with the rural ED physician. The patient is transferred into the MSU for an urgent brain CT scan, with images being transmitted to the UAH tele-stroke neurologist for review in real-time. The stroke fellow completes the initial evaluation of the patient. The UAH telestroke neurologist via a secure telemedicine link evaluates the neurological examination with the stroke fellow and confirms the diagnosis. The diagnosis may be an acute stroke that requires intravenous thrombolysis or several other medical conditions. These include transient ischemic attacks (TIAs), mild stroke that do not require thrombolysis and very frequently stroke ‘mimics’. There are two management options available: Treatment with IV rt-PA and transport to UAH for further management or supportive care and transport to UAH if necessary (option #1; Triage to CSC), or transfer back to the local referring hospital following imaging and consultation in the MSU (Option #2; Triage to LHC). The option to repatriate the patient back to the referring hospital is most frequently entertained in cases where the diagnosis includes transient ischemic attack (TIAs), minor strokes, malignant ischemic stroke or intracerebral hemorrhage (ICH) with advanced patient directive for palliative care, and stroke mimics. The decision to ship to the UAH or repatriate to the home hospital is at the discretion of the tele-stroke neurologist. All patients admitted to UAH or home hospital with acute stroke get standard of care according to the Canadian Stroke Best Practices guidelines^11^.

### Follow up

Once the patients are discharged from UAH or home hospital, they are assessed in stroke prevention clinics vis direct visit or telestroke consult within the first 3 months of symptom onset. The vascular risk factor control targets and etiological evaluation are communicated and completed with the help of the Primary Care Provider.

### Statistical analysis

The patients were divided into two groups, patient transported to comprehensive stroke center for further treatment (Triage to CSC Group) and patients transported back to the home hospital for further management (Triage to LHC Group). The continuous variables with non-skewed distribution including age, systolic and diastolic blood pressure were expressed as mean with standard deviation. Continuous variables with skewed distribution including dispatch to patient duration, symptom onset to patient duration, symptom onset to CT duration, patient to CT duration, NIHSS, and modified Rankin scale were expressed as median (Interquartile range). Nominal variables including risk factors, sex-distribution, stroke subtype, recurrent stroke and mortality were expressed as numbers and percentages. Differences in continuous variables were assessed by t-test, and Mann–Whitney U test for medians, and Fischer exact test and Chi-Square test for catergorical variables. All statistical analysis was performed on IBM SPSS Statistics version 26 (IBM, Armonk, USA).

### Ethics approval

The University of Alberta Ethics Review Committee approved the project.

## Data Availability

Data will be available on request. Please email the corresponding author via email.

## References

[1] Kunz A (2017). Effects of ultraearly intravenous thrombolysis on outcomes in ischemic stroke: The STEMO (Stroke Emergency Mobile) Group. Circulation.

[2] Kim, J.-T. *et al.* Treatment with tissue plasminogen activator in the golden hour and the shape of the 4.5-hour time-benefit curve in the national United States get with the guidelines-stroke population. *Circulation* **135**, 128–139 (2017). 10.1161/CIRCULATIONAHA.116.023336 27815374

[3] Calderon VJ, Kasturiarachi BM, Lin E, Bansal V, Zaidat OO (2018). Review of the mobile stroke unit experience worldwide. Interv. Neurol..

[4] Helwig SA (2019). Prehospital stroke management optimized by use of clinical scoring vs mobile stroke unit for triage of patients with stroke: A randomized clinical trial. JAMA Neurol..

[5] Mathur S (2019). Improving prehospital stroke services in rural and underserved settings with mobile stroke units. Front. Neurol..

[6] Zhao, H. *et al.* Greater clinical impact of thrombectomy than thrombolysis. 922–930, 10.1161/STROKEAHA.119.027843 (2020). 10.1161/STROKEAHA.119.027843 32078483

[7] Weber JE (2013). Prehospital thrombolysis in acute stroke: Results of the PHANTOM-S pilot study. Neurology.

[8] Gioia, L. C. *et al.* Prehospital systolic blood pressure is higher in acute stroke compared with stroke mimics. *Neurology* **86** (2016). 10.1212/WNL.0000000000002747 PMC4898317 27194383

[9] Campbell BCV (2015). Endovascular therapy for ischemic stroke with perfusion-imaging selection. N. Engl. J. Med..

[10] Itrat A (2016). Telemedicine in prehospital stroke evaluation and thrombolysis taking stroke treatment to the doorstep. JAMA Neurol..

[11] Boulanger, J. M. *et al.* Canadian stroke best practice recommendations for acute stroke management: Prehospital, Emergency Department, and Acute Inpatient Stroke Care, 6th edition, update 2018. *Int. J. Stroke* **13**, 949–984 (2018). 10.1177/1747493018786616 30021503

[12] Parker SA (2015). Establishing the first mobile stroke unit in the United States. Stroke.

[13] Bowry R (2015). Benefits of stroke treatment using a mobile stroke unit compared with standard management: The BEST-MSU study run-in phase. Stroke.

[14] Wendt M (2015). Improved prehospital triage of patients with stroke in a specialized stroke ambulance: Results of the pre-hospital acute neurological therapy and optimization of medical care in stroke study. Stroke.

[15] Walter S (2012). Diagnosis and treatment of patients with stroke in a mobile stroke unit versus in hospital: A randomised controlled trial. Lancet Neurol..

[16] Sablot, D. *et al.* Complications during inter-hospital transfer of patients with acute ischemic stroke for endovascular therapy. *Prehospital Emerg. Care* 1–7, 10.1080/10903127.2019.1695299 (2020). 10.1080/10903127.2019.1695299 31750753

[17] Ebinger M (2014). Effect of the use of ambulance-based thrombolysis on time to thrombolysis in acute ischemic stroke: A randomized clinical trial. JAMA J. Am. Med. Assoc..

[18] Hansson, P. O., Andersson Hagiwara, M., Herlitz, J., Brink, P. & Wireklint Sundström, B. Prehospital assessment of suspected stroke and TIA: An observational study. *Acta Neurol. Scand.* **140**, 93–99 (2019). 10.1111/ane.13107 31009075

[19] Geisler F (2019). Evaluation of a score for the prehospital distinction between cerebrovascular disease and stroke mimic patients. Int. J. Stroke.

[20] Simhan, S. *et al.* The outcome of acute functional neurological disorder: A meta-analysis of stroke-mimic presentations. *J. Neurol.* 1–5, 10.1007/s00415-020-09709-3 (2020). 10.1007/s00415-020-09709-3 31970491

[21] Schwindling L (2016). Prehospital imaging-based triage of head trauma with a mobile stroke unit: First evidence and literature review. J. Neuroimaging.

[22] Shuaib A, Jeerakathil T (2018). The mobile stroke unit and management of acute stroke in rural settings. CMAJ.

[23] Shuaib A (2018). Mobile stroke unit triage of patients with a suspected stroke: A novel solution to reducing suspected stroke admissions in busy emergency departments. BMJ Innov..

